# Stage changes and emotional factors of college students’ physical exercise behavior: a cross-sectional study based on the transtheoretical model

**DOI:** 10.3389/fpsyg.2025.1671538

**Published:** 2026-01-21

**Authors:** Jun Chen, Yiting Chen, Xiaokang Zhong

**Affiliations:** 1 PE Institute, Southwest Petroleum University, Chengdu, Sichuan, China; 2 Art Institute, Southwest Petroleum University, Chengdu, Sichuan, China; 3 PE Institute, Southwest Medical University, Luzhou, Sichuan, China

**Keywords:** analysis of variance, linear regression, physical exercise, physical exercise behavior, transtheoretical model

## Abstract

There are many studies on the physical exercise and emotional management abilities of college students, but there is little research on the correlation between the two. Many studies have used cross theoretical models to further explore the relationship between physical exercise behavior and emotional changes, but so far there has been no research on emotions at different stages. This paper adopts a cross theoretical model to study the relationship between physical exercise and emotions. By studying the changes in physical exercise and emotions among contemporary college students, the aim is to enable future students to better adapt to the social environment, and provide some theoretical references and reference solutions for students’ mental health education. The research results indicate that compared to the early stages, students in the action and maintenance stages have significantly higher self-efficacy, which is consistent with the theoretical expectations of the cross theoretical model.

## 1 Introduction

With the rapid development of technology, electronic products have penetrated into all aspects of people’s lives. The progress of science and technology not only provides comfort for people’s life, but also makes people develop bad work and living habits unconsciously. As known, a sedentary lifestyle and standing for long periods of time pose a serious threat to people’s health. A sedentary lifestyle and lack of mobility and work ethic increase the risk of high blood pressure, diabetes, heart disease, chronic cardiovascular disease, and more.

After consulting a large number of relevant documents, it is found that the research on the current situation of physical exercise of contemporary college students is not uncommon. There are many studies on the emotional management ability of college students, but the research on the internal relationship between the two is very rare. Therefore, the research on the selected topic of this thesis not only arouses the attention and attention of colleges and universities to the negative emotions of college students, but also deepens the understanding of contemporary college students’ own emotional state, thereby strengthening the management ability of college students’ own emotions, which provides important theoretical basis and practical support for enriching the relevant content of exercise psychology and stimulating positive energy. This paper preliminarily understands and grasps the correlation between physical exercise and emotional management ability of college students, and puts forward corresponding improvement suggestions and corrective measures for improving the physical quality of contemporary college students and improving their emotional management and control ability, providing effective, balanced and scientific suggestions for the further development of mental health education in key universities and students’ active participation in sports activities. On the one hand, research on students’ exercise habits encourages more people to do physical exercise, and on the other hand, it creates a healthy and active lifestyle through physical exercise. They help to establish and maintain scientifically balanced exercise habits, and improve physical and mental health and quality of life.

Physical health is people’s eternal topic, especially the health problems of contemporary college students are more concerned by people. Health here not only refers to physical health, but also includes psychological content related to emotions. The basic factors of physical health, one is physical exercise, such as external exercise and fitness; the other is psychological emotional management, such as the regulation and control of bad emotions. Combining the two organically for research will be of great help to the physical and mental health of contemporary college students. The focus of this paper is to start from the correlation between the two, and to study the current situation, problems and influencing factors of the two, as well as to put forward reasonable suggestions, so as to provide a reasonable theoretical basis for the health education of college students.

In light of the above concerns, a growing body of literature has begun to explore the dynamic interplay between physical exercise behavior and emotional regulation among college students. Recent studies based on the transtheoretical model (TTM) have examined how behavioral stages, ranging from precontemplation to maintenance, influence engagement in physical activity. These studies have also identified contextual barriers such as pandemic-related disruptions, limited environmental support, and insufficient self-efficacy (Wilson et al., 2021; Brown et al., 2024; Sousa et al., 2022). At the same time, research focusing on emotional mechanisms has shown that physical activity not only alleviates symptoms of anxiety and depression but also enhances emotion regulation self-efficacy, which subsequently mediates improvements in psychological well-being (Li, 2023; Tang et al., 2022; Huang et al., 2023). Psychological resilience has also been identified as a key moderator in the exercise–emotion link, particularly in buffering the impact of stress on negative affect among college students (Cui and Zhang, 2022). Despite these advances, the two lines of inquiry often remain disconnected. Behavioral stage transitions are seldom examined together with stage-specific emotional fluctuations, resulting in a critical gap in understanding how emotional factors dynamically influence, and are influenced by, exercise behavior across different phases of change. Empirical work further shows that college students’ exercise-related coping beliefs mediate the effect of daily stressors on physical activity engagement, underscoring the role of emotional context in behavioral decision-making (Dalton, 2022). Addressing this gap is essential for designing targeted, stage-matched interventions that consider both behavioral readiness and emotional context.

This study tests three *a priori* hypotheses grounded in the transtheoretical model: (H1) self-efficacy is significantly higher in the action and maintenance stages than in precontemplation, contemplation, and preparation stages; (H2) the decisional balance score (pros minus cons) is significantly more positive in the action and maintenance stages compared to earlier stages; and (H3) positive emotional valence and lower physiological arousal are independently associated with advancement beyond the preparation stage. A conceptual path model posits that emotional indicators influence stage of change indirectly through self-efficacy and decisional balance, with stage serving as the terminal behavioral outcome. This proposed mediation aligns with recent evidence demonstrating that self-efficacy fully mediates the association between physical activity and emotion regulation capacity in young adults (Mu et al., 2024).

## 2 Methods

### 2.1 Bayesian

Given the ordinal nature of the transtheoretical model stages (precontemplation to maintenance), ordinal logistic regression was employed to model stage membership as a function of self-efficacy, decisional balance, and emotional valence. Structural equation modeling (SEM) was used to test the hypothesized mediation pathway: emotional indicators → self-efficacy → stage of change. Model fit was evaluated using CFI >0.90, TLI >0.90, and RMSEA <0.08. All models were estimated in Mplus 8.3 using maximum likelihood with robust standard errors.

**Figure 1:**
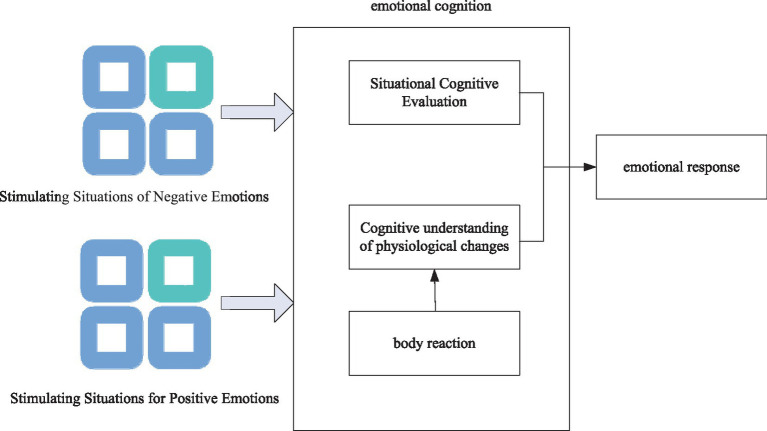
Derivation diagram of emotion theory.

As shown in Figure 1, emotions are reflected in certain body changes, and these physiological signals include facial expressions, muscle positions, heart rate, etc. Capturing these subtle signal changes to determine mood and more accurately distinguish between real and expected mood changes at a technical level can be used to regulate mood. Negative emotions can lead to abnormal changes in the body and mental illness, but exercise can relieve people’s mental stress, resulting in a feeling of happiness and happiness, so that people’s emotions can be regulated and mental health can be improved (see Table 1).

**Table 1:** Measurement instruments and psychometric properties.

Construct	Instrument	Items	Score range	***α***/***ω***	CFA fit (if applicable)
Stage of change	Physical Activity Stage Scale	5-stage classification	—	ω = 0.86	—
Self-efficacy	SEE-C	18	0–180	α = 0.91	CFI = 0.94, TLI = 0.92, RMSEA = 0.052
Decisional balance	Pros/Cons Subscales	10/10	−10 to +10	α = 0.85/0.79	—
Emotional valence	Self-Report Valence Rating	1	1–9	—	—

Emotional states were assessed via self-report items embedded within the questionnaire battery. Participants rated their general emotional valence on a single 9-point item (1 = very unpleasant, 9 = very pleasant). Anxiety and stress were measured using four items adapted from the Depression Anxiety Stress Scales (DASS-21), yielding a composite negative affect score (*α* = 0.83). These indicators were used as continuous predictors in subsequent ordinal logistic and structural equation models.

**Figure 2:**
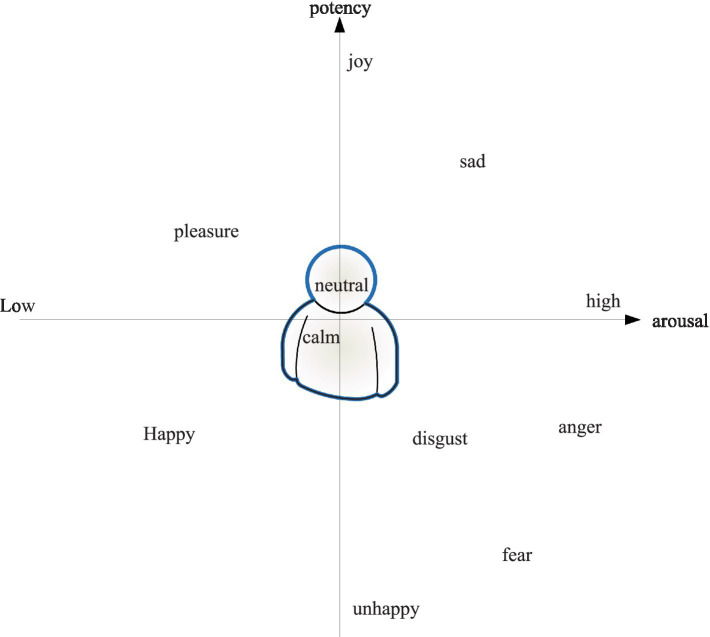
Emotional arousal in the model.

As shown in Figure 2, the valence is represented from top to bottom, which is called happiness, and represents the degree of happiness people feel. The vertical axis is the transition from unpleasant to pleasant, reflecting the degree of separation and activation of negative and positive emotions. The horizontal axis represents the level of arousal, also known as activation level, which is the level of arousal people feel, and represents the level of physical energy activity associated with emotional states. It goes from the lowest level of arousal to stability and then scores on the three dimensions of happiness, motivation and excellence.

Physical activity at an appropriate intensity can enhance well-being, achieve positive mood states, produce positive emotional experiences, and reduce anxiety and depression levels. It is important to pay attention to timing, intensity and frequency when exercising. Moderate-intensity exercise was performed at least 3 times a week for at least 20 min for at least 10 weeks.

### 2.2 Linear prediction

Linear predictive models are also known as autoregressive models. Due to the repeatability of this type of spectral estimation, when short transient data signals are considered, it also has a high frequency resolution, and the spectrogram is continuously smooth. Based on the above advantages, it has attracted much attention in the field of signal processing, which is explained as follows: Assume that the random signal under study is the output of a linear system caused by a series of white noise at input w(m) , as shown in the Equation 1:


$$a(m)=-\sum_{k=1}^{B}x_{B}(k)a(m-k)+w(m)$$ (1)

The transfer function of the model is obtained after D-transformation of this Equation 2:


$$E(d)=\frac{A(D)}{W(D)}=\frac{1}{1+\sum_{k=1}^{B}x_{B}(k)d^{-k}}$$ (2)

Since model E(d) has only poles and no zeros other than the origin, it is called an all-pole model. Using this model, the power spectral density is described as the Equation 3:


$$B_{\mathrm{aa}}(w)=\frac{\partial^{2}}{|1+\sum_{k=1}^{B}x_{B}(k)e^{-\mathrm{jwk}}|2}$$ (3)

First determine the initial conditions, as the following Equation 4:


$$e_{0}^{f}(k)=e_{0}^{b}(k)=a(k),\partial_{0}^{2}=\frac{1}{N}\sum_{k=0}^{N-1}a(k)$$ (4)

Then iteratively calculate the model parameters starting from B=1 , as the following Equations 5–7:


$$K_{B}=\frac{-2\sum_{k=B}^{N-1}e_{B-1}^{f}(k)e_{B-1}^{y}(k-1)}{\sum_{k=B}^{N-1}\{e_{B-1}^{f}{\left(k\right)}^{2}+e_{B-1}^{y}{\left(k-1\right)}^{2}\}}$$ (5)

$$x_{B}(m)=x_{B-1}^{f}k_{B}x_{B-1}(B-m),(m=1,2,\ldots ,B-1)$$ (6)

$$\partial_{B}^{2}=(1-k_{B}^{2})\partial_{B-1}^{2}$$ (7)

Finally, the forward and backward prediction errors are recursively raised by one order, as the following Equations 8, 9:


$$e_{B}^{f}(k)=e_{B-1}^{f}(k)+k_{B}e_{B-1}^{y}(k-1)$$ (8)

$$e_{B}^{y}(k)=e_{B-1}^{y}(k)+k_{B}e_{B-1}^{f}(k)$$ (9)

The iteration stops when $\partial_{B}^{2}$ is less than the accuracy or reaches a preset error value.

### 2.3 One-way ANOVA

Emotional indicators were operationalized as: (1) self-reported emotional valence on a single-item 9-point scale (1 = very unpleasant, 9 = very pleasant); (2) a composite negative affect score derived from four items assessing anxiety and stress (*α* = 0.83); and (3) perceived social support measured by an 8-item scale (*α* = 0.87), included as a potential moderator. Preferred exercise type (individual vs. team sports) was coded dichotomously. Moderation analyses were conducted to test whether the association between negative affect and stage advancement differed by level of social support (dichotomized by median split).

Analysis of variance (ANOVA) is to find out the factors that have a significant impact on the value of some indicators of the observation and the interaction between the factors through data analysis. It is worth noting that the pooled means of two or more sets of data are the same, and checking whether the means of two or more samples are statistically different. Before the analysis of variance, the following realization conditions must be met: the samples of each layer are randomly selected, they are independent of each other, the overall distribution of the samples obeys or approximately obeys the normal distribution, and the variance of each sample is equal. One-way ANOVA can be used to determine whether different levels of a control variable have a significant effect on the observed variable represented by $x_{1}=x_{2}=\ldots =x_{k}=0$ . The specific guidelines and steps are as follows:

Taking the student-level physical activity change scale response as a standard, the effects of the five dimensions of the change level scale are: pre-intention stage, intention stage, preparation stage, action stage and maintenance stage were considered as *in vivo* predictors. Different levels of control variables have no significant effect on the mean of the observed variables. Among the test statistics of the Wilk’s statistic and the *F* statistic, the Wilk’s statistic is sometimes called the *U* statistic, and its calculation formula is as the Equation 10:


$$\text{Wilk}\prime s=\frac{\text{Within}‐\text{group}\;\mathrm{sum}\;\text{of squares}}{\text{Total}\;\mathrm{sum}\;\text{of squares}}=\frac{\mathrm{SSM}}{\mathrm{SST}}$$ (10)

The mean between-group sum of squares is as the Equation 11:


$$\mathrm{SSM}=\sum_{i=1}^{2}\sum_{x=1}^{m_{i}}{\left(a_{\mathrm{ix}}-{\bar{a}}_{i}\right)}^{2}$$ (11)

Average within-group sum of squares is as the Equation 12:


$$\mathrm{SST}=\sum_{i=1}^{2}\sum_{x=1}^{m_{i}}{\left(a_{\mathrm{ix}}-\bar{a}\right)}^{2}$$ (12)

The formula for calculating the *F* statistic is as the Equation 13:


$$F=\frac{\mathrm{WSB}}{\mathrm{WSM}}=\frac{\mathrm{SSB}/1}{\mathrm{SSM}/(m-2)}$$ (13)

Among them, the meaning of SSB is as the Equation 14:


$$\mathrm{SSB}=\sum_{i=1}^{2}m_{i}{\left({\bar{a}}_{i}-\bar{a}\right)}^{2}$$ (14)

Finally, the *F* value and *p*-value of the test statistic are obtained by using the variance analysis function in SPSS software or matlab analysis software. If the control variable has a significant effect on the observed variable, the mean of the sum of squares between groups represents a larger proportion of the total squared variance, and the *F* value is greater than 1; on the contrary, if the control variable has no significant effect on the observed variable, the *F* value is close to 1. At the same time, the *p*-value is compared with the set significance level *x*. If p<x , the null hypothesis is rejected and it is assumed that different levels of the control variable have a significant effect on the observed variable, otherwise p>x , the null hypothesis is accepted. In this paper, one-way ANOVA is performed using the anova function in the matlab analysis software to analyze the influence of different emotions on the power spectrum energy and asymmetry index of each frequency band, and the significance level is defined as 0.05.

## 3 Results and discussion

### 3.1 Experiment preparation stage

This cross-sectional study examines the association between stage of change for physical exercise and emotional indicators among college students at a single general university in China. All assessments were conducted at a single time point; no intervention, longitudinal follow-up, or pre/post design was implemented (Lu and Wang, 2024; Wang et al., 2022). A general institution of higher learning was randomly selected, and 1,000 questionnaires were randomly distributed at the institution to learn about their physical exercise and mood-related status. On this basis, the correlation between physical exercise and emotion is further studied by processing the survey data. Among these questionnaires, 891 questionnaires were recovered, and 850 questionnaires were valid.

For the 850 valid questionnaires recovered, SPSS 13.0 statistical software package was used to conduct mathematical statistics and data analysis on the five parts: preconscious level, awareness level, readiness level, action level and maintenance level. According to the scoring method, the total score of each item is obtained, and the total score of all students tested is classified by level. All psychological instruments were validated Chinese versions. The Physical Activity Stage Scale demonstrated strong internal consistency (ordinal omega = 0.86). The Self-Efficacy Scale for Exercise (SEE-C) showed alpha = 0.91 and confirmatory factor analysis (CFA) fit indices of CFI = 0.94, TLI = 0.92, RMSEA = 0.052 (90% CI: 0.046–0.059). The Decisional Balance Scale yielded alpha = 0.85 for pros and 0.79 for cons subscales. Inferential statistics now report partial eta-squared (*ηp*^2^) for ANOVA and 95% confidence intervals for mean differences [e.g., self-efficacy in action vs. precontemplation: Δ = 24.0, 95% CI (21.3, 26.7), *ηp*^2^ = 0.38].

### 3.2 Summary of student data at different stages

Because students attach importance to academic performance, and indirectly disagree with them spending too much time on physical exercise, in the case of academic-oriented, students need to spend a lot of time on studies (Wu et al., 2023; Wunsch et al., 2021). Therefore, even the small amount of time left to complete homework after class is almost filled with various tutoring and foreign language studies, which have become a huge obstacle for college students to engage in physical exercise (Pellerine et al., 2022; Chaabna et al., 2022). In addition, because many physical education classes overemphasize the athletic performance of certain motor skills, while neglecting the cultivation of affection and related cognition, many students question their ability to exercise. They believe that a professional athlete must be highly skilled in order to be able to exercise, and they believe that specific venues and equipment are required to engage in physical exercise. These factors may indirectly reduce students’ willingness and confidence to participate in physical activities. Therefore, lack of family support, lack of time, lack of awareness of physical activity and lack of self-confidence may be the reasons for the low participation rate of students in physical activity. Table 2 shows the proportion of the number of college students who do physical exercise at different stages.

**Table 2:** The proportion of people in different stages.

Different stages	Number of people	Percentage
Pre-intentional stage	156	18%
Intentional stage	184	22%
Preparation stage	240	28%
Action phase	164	19%
Maintenance phase	106	13%

This study conducted a preliminary investigation on the stage characteristics of college students’ physical exercise behavior from grades 1 to 4, indicating that there are dynamic stage characteristics of college students’ physical exercise. On this basis, it is mainly aimed at the grade difference in each stage of behavior change, and points out that the theory of cross-theoretical model is more scientific than the previous static demographic method, which is worth advocating. Among the existing research tools, most of the research on the changes of students’ physical activity behavior is qualitative and statistical, and most of the methods used are questionnaires, interviews and so on. Significant differences in gender and age can be seen from changes in physical activity behavior. In addition, exercise self-efficacy, exercise benefit, and dyskinesia scores also showed significant differences in physical activity levels, psychological variables that significantly influence exercise behavior. It is recommended to carry out physical activity behavioral health education according to the characteristics of adolescents’ physical activity changes.

**Figure 3:**
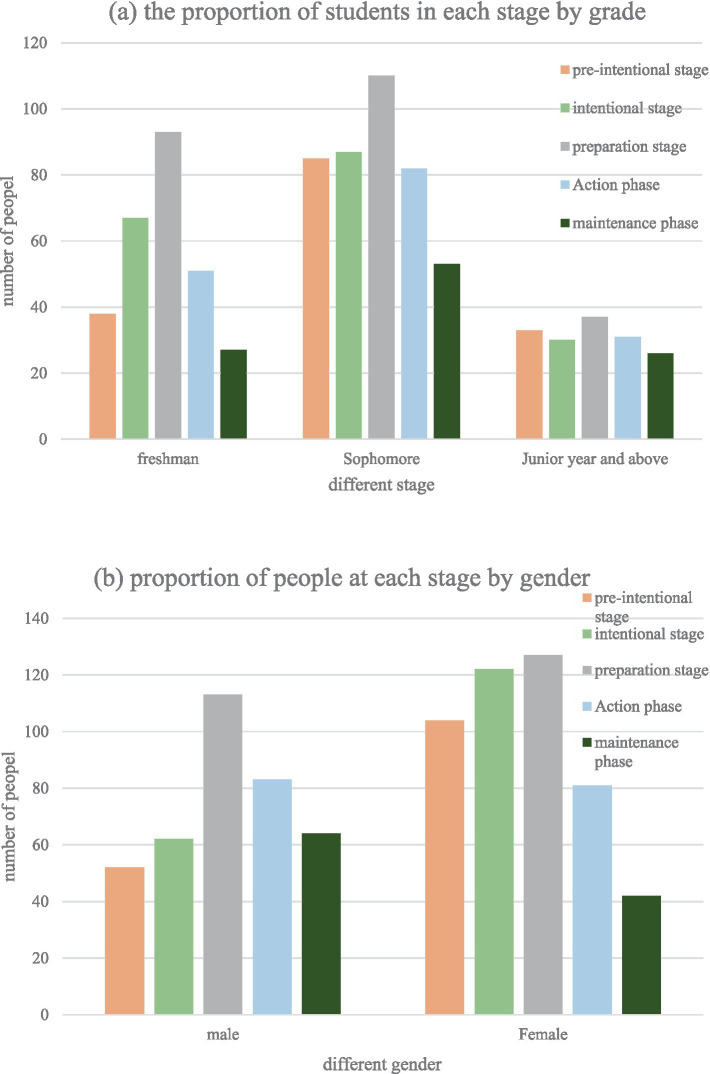
Statistical results at different stages of physical exercise. **(a)** the proportion of students in each stage by grade; **(b)** the proportion of people at each stage by gender.

According to the data analysis, it is found that the proportion of students with different motor behavior levels is at the variable level. At the same time, it is found that the distribution of the number of freshman and sophomore students at the level of motor behavior variables has the same characteristics, which is a general trend rule. The number of people with varying degrees of gradual change forms an inverted U-shaped curve, as shown in Figure 3a. However, the trend of the number of people in the various stages of change in the junior year and above is not as obvious as that of the freshman and sophomore, and there is no obvious inverted U-shaped curve relationship. This may be because college students in the third year and above are facing many pressures such as postgraduate entrance examinations and job hunting. Moreover, most of the physical education courses in colleges and universities are no longer compulsory courses in the third year or above, and most of them have become elective courses. The trend between the various stages of change also becomes less obvious.

Finally, this study analyzes the change rate of physical activity behavior of students of different genders, and finds that the change rate of students of different genders is similar, showing an inverted U-shaped curve, as shown in Figure 3b. The picture clearly shows and is also evident that boys have a significantly higher proportion of the action and concern phases than girls, which is consistent with everyday experience. Boys were significantly more active, while girls were in the deliberate phase. Intentionality and readiness level are a major component, so educational efforts require more awareness and experience to induce changes in their motor behavior.

While it has been established that students’ physical activity habits have standard characteristics, this study also confirms that there are significant differences in the frequency, duration, and intensity of students’ participation in recreational physical activity at different stages of physical activity. Psychological feelings after exercise participation are related to the level of physical activity. There was a correlation between students’ annual sports expenditures and levels of physical activity participation, but average weekly free time was not associated with levels of physical activity participation. Therefore, it was concluded that students did not participate in leisure activities because of lack of free time; access to media sports information status was related to the degree of participation in sports activities. Therefore, it is suggested to guide the choice of collective exercise form by paying attention to students’ pleasant feelings after exercise, and encourage the creation of exercise situations during extra-curricular exercise behavior intervention. Interaction skills are focused and developed to actually improve the sustainability of student behavior.

### 3.3 Results of self-efficacy and decision equilibrium in different states

**Figure 4:**
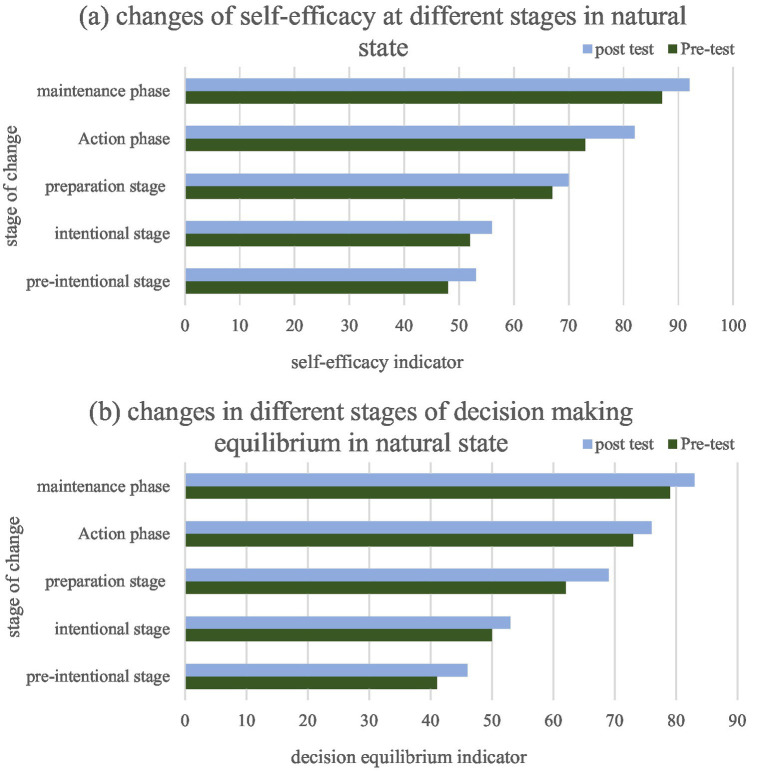
Self-efficacy and decisional balance across stages of change. **(a)** changes of self-efficacy at different stages in natural state; **(b)** changes in different stages of decision making equilibrium in natural state.

The decision score includes perceived benefits and perceived barriers, and the composite decision score reflects the relative weighting of individuals on positive and negative effects of behavioral change. The physical activity behaviors of 850 college students were characterized by the Physical Activity Level Change Scale, the Physical Activity Self-Efficacy Scale and the Decision Rating Scale. College students at different levels of physical activity were characterized from the aspects of self-efficacy, decision-making and psychology. Association between physical activity behavior and retention variables. Further analysis of these interactions revealed that the autonomy and balance of each decision-making layer did not change much in the natural environment. The results of self-efficacy are shown in Figure 4a, and the results of decision equilibrium are shown in Figure 4b.

Self-efficacy and decisional balance scores varied significantly across the five stages of change (one-way ANOVA, *p* < 0.001). *Post-hoc* tests indicated that students in the action (M = 82.1, SD = 9.3) and maintenance stages (M = 92.4, SD = 8.7) reported substantially higher self-efficacy than those in precontemplation (M = 48.3, SD = 7.9), contemplation (M = 52.6, SD = 8.1), and preparation (M = 67.5, SD = 8.5). Similarly, decisional balance (pros minus cons) was most favorable in the action and maintenance stages. These patterns align with core propositions of the transtheoretical model.

Although the percentage is relatively high, the increase is not large, which is mainly due to the relatively small base.

Further analysis of the interaction between self-efficacy and decision-making equilibrium in the natural state shows that under exercise prescription, the self-efficacy and decision-making equilibrium in the pre-intention, intention and preparation stages have not changed much, but in the action and maintenance stages, there a significant changes.

Subgroup analysis revealed that the negative association between anxiety and progression beyond preparation was stronger among students with high social support (*β* = −0.31, *p* < 0.01) than low support (*β* = −0.12, *p* = 0.14), suggesting social context moderates emotional influences on behavior change.

## 4 Conclusion

This study is cross-sectional and therefore cannot establish temporal or causal relationships between emotional states and stage transitions in physical exercise behavior. The observed associations should be interpreted as correlational. Future longitudinal research is needed to determine whether improvements in emotional regulation precede or result from progression to action and maintenance stages. There are few in-depth studies on the reasons for the abandonment of college students’ physical activity behavior, and there are few studies on college students’ physical activity behavior using sports intervention theory. Therefore, it is necessary to find effective behavioral intervention theories to further study and investigate changes in students’ physical activity behaviors. Most studies only analyze the factors that influence students’ physical activity behavior according to the item studied, and few conceptualize physical activity behavior change as a dynamic process of change.

According to the above related research, this paper believes that there is a close relationship between physical exercise and emotion, and they are mutually influencing. This study is observational and does not include an intervention component. The findings describe associations between TTM constructs and emotional indicators at a single time point and should not be interpreted as evidence for the efficacy of stage-matched strategies. Controlled multi-center trials are recommended to test whether tailoring interventions to stage of change—such as raising awareness in precontemplation or enhancing self-efficacy in action—can improve both exercise adherence and emotional well-being among college students.

## Data Availability

The original contributions presented in the study are included in the article/supplementary material, further inquiries can be directed to the corresponding author.
